# The Impact of Accelerated Right Prefrontal High-Frequency Repetitive Transcranial Magnetic Stimulation (rTMS) on Cue-Reactivity: An fMRI Study on Craving in Recently Detoxified Alcohol-Dependent Patients

**DOI:** 10.1371/journal.pone.0136182

**Published:** 2015-08-21

**Authors:** Sarah C. Herremans, Peter Van Schuerbeek, Rudi De Raedt, Frieda Matthys, Ronald Buyl, Johan De Mey, Chris Baeken

**Affiliations:** 1 Department of Psychiatry, Vrije Universiteit Brussel (VUB), Universitair Ziekenhuis Brussel (UZ Brussel), Brussels, Belgium; 2 Department of Radiology, Vrije Universiteit Brussel (VUB), Universitair Ziekenhuis Brussel (UZ Brussel), Brussels, Belgium; 3 Department of Experimental Clinical and Health Psychology, Ghent University, Ghent, Belgium; 4 Department of Biostatistics and Medical Informatics, Vrije Universiteit Brussel (VUB), Brussels, Belgium; 5 Department of Psychiatry and Medical Psychology, Ghent University, Universitair Ziekenhuis Gent, Ghent, Belgium; University of Regensburg, GERMANY

## Abstract

In alcohol-dependent patients craving is a difficult-to-treat phenomenon. It has been suggested that high-frequency (HF) repetitive transcranial magnetic stimulation (rTMS) may have beneficial effects. However, exactly how this application exerts its effect on the underlying craving neurocircuit is currently unclear. In an effort to induce alcohol craving and to maximize detection of HF-rTMS effects to cue-induced alcohol craving, patients were exposed to a block and event-related alcohol cue-reactivity paradigm while being scanned with fMRI. Hence, we assessed the effect of right dorsolateral prefrontal cortex (DLPFC) stimulation on cue-induced and general alcohol craving, and the related craving neurocircuit. Twenty-six recently detoxified alcohol-dependent patients were included. First, we evaluated the impact of one sham-controlled stimulation session. Second, we examined the effect of accelerated right DLPFC HF-rTMS treatment: here patients received 15 sessions in an open label accelerated design, spread over 4 consecutive days. General craving significantly decreased after 15 active HF-rTMS sessions. However, cue-induced alcohol craving was not altered. Our brain imaging results did not show that the cue-exposure affected the underlying craving neurocircuit after both one and fifteen active HF-rTMS sessions. Yet, brain activation changes after one and 15 HF-rTMS sessions, respectively, were observed in regions associated with the extended reward system and the default mode network, but only during the presentation of the event-related paradigm. Our findings indicate that accelerated HF-rTMS applied to the right DLPFC does not manifestly affect the craving neurocircuit during an alcohol-related cue-exposure, but instead it may influence the attentional network.

## Introduction

Alcohol addiction is a chronic relapsing disorder. A possible contributing factor to relapse is alcohol craving, even when alcohol-dependent patients are effectively detoxified [1]. Craving is defined as a subjective experience of wanting to use drugs [2]. Tiffany (1990) [3] stated that conscious craving, which may be triggered by conditioned drug cues, only occurs when the automatic process of drug intake is interrupted. According to Grüsser et al (2004) [4], non-conscious appetitive reactions occur when patients are confronted with alcohol-related stimuli. These reactions might explain why alcohol-dependent patients relapse even though they do not always acknowledge experiencing craving [5].

The rewarding effects of alcohol and drugs of abuse are mediated by the mesocorticolimbic system that consists of the ventral tegmental area (VTA), nucleus accumbens (NcAc), amygdala and prefrontal cortex [6]. Because of their role in processing salience and reward information in cue- and drug-induced craving, Koob and Volkow (2010) [7] emphasize the implication of the orbitofrontal (OFC), medial prefrontal (MPFC) and the anterior cingulate cortex (ACC). This is consistent with existing cue-induced functional brain activation studies, which further demonstrate the implication of the ventral striatum (includes the Nucleus Accumbens, NcAc), the dorsal striatum (consists of the caudate nucleus and putamen), and the basolateral amygdala in cue-reactivity [8]. Because of its involvement in reward evaluation, motivation, attention, and flexibility, also the DLPFC is an important component of the brain reward circuitry [7,9].

To treat alcohol craving few pharmacotherapeutic options are currently available (of which only naltrexone and acamprosate are FDA approved) [10]. The scarce existing studies show that especially naltrexone is able to influence orbitofrontal cortical processes, implicated in the underlying craving neurocircuit in alcohol-dependent patients [11,12]. However, larger studies have to substantiate these findings.

As a new neuroscience tool in addiction, the application of repetitive transcranial magnetic stimulation (rTMS), a non-invasive neurostimulation technique, has focused mostly on subjective craving and to some extent on cognitive processes [13,14]. With regard to craving effects, until now only DLPFC rTMS has been investigated [15]. Notwithstanding that Herremans et al (2012) [16] could not demonstrate a decrease in alcohol craving after one right DLPFC high-frequency (HF)-rTMS session, Mishra and colleagues (2010) [17] found a beneficial effect of ten daily sessions of right DLPFC HF-rTMS on subjective alcohol craving measurements.

Höppner et al (2011) [18] did not find any attenuation of craving scores when alcohol-dependent patients were administered left DLPFC HF-rTMS stimulation. In contrast, Mishra et al (2015) [19] showed a beneficial effect on alcohol craving for both left and right DLPFC stimulation. However, the application of HF-rTMS has been evaluated in other substance dependencies as well. In nicotine research, left DLPFC stimulation resulted in a decrease of nicotine craving [20]; whereas left and right DLPFC stimulation resulted in a decrease of cocaine craving [21,22]. Nevertheless, in addition to these contradicting clinical findings, to date, the underlying mechanism of action of left or right HF-rTMS on alcohol craving in addiction remains poorly understood.

Consequently, our major objective was to further evaluate the underlying neuronal mechanisms of action of right DLPFC HF-rTMS stimulation on alcohol craving in recently detoxified hospitalized patients. As previous research already demonstrated that craving intensifies when alcohol-dependent patients are confronted with alcohol-related stimuli [23], to maximize the detection of HF-rTMS effects, patients were subjected to two alcohol-related cue-exposures during fMRI, a block and event-related paradigm. We decided to evaluate two distinct fMRI paradigms because at this moment, it is unclear which may provide the most useful information on the neurocircuit of craving. Blocked paradigms have an adequate signal-to-noise ratio leading to an increase in statistical power. They also provide a relatively larger BOLD signal change related to baseline. However, advantages of using an event-related cue-exposure are the possibility to detect transient variations in hemodynamic responses and to maintain patients' attentional level across the experiment [24]. Even though the DLPFC is not majorly involved in cue-reactivity in treatment-seeking alcohol-dependent patients [25] previous research shows that DLPFC HF-rTMS stimulation is able to 1) decrease craving in substance addiction [26,27], and 2) influence ACC activity, which is part of the craving neurocircuit [28,29]. Because former studies mostly demonstrated a reduction of alcohol craving after right-sided stimulation, we targeted the right DLPFC.

The current fMRI study consisted of 2 distinct research questions: an experimental part—evaluating one placebo-controlled HF-rTMS stimulation session—and an open label treatment part, consisting of fifteen accelerated active HF-rTMS sessions spread over 4 days. Such accelerated HF-rTMS protocols, where multiple sessions on the same day are applied, have already been evaluated in depressed patients, suggesting this may have the potential to result in faster clinical responses [30,31]. In craving research patients may only benefit when multiple HF-rTMS sessions are applied [16] and although such an intensive protocol shortens the treatment duration considerably and could accelerate craving reductions, no such studies are performed yet.

Before stimulation, we expected that both cue-exposure paradigms would provoke immediate craving and elicit brain activation alterations especially in the craving neurocircuit. For the sham-controlled single HF-rTMS session, we expected to find that only active and not sham HF-rTMS would suppress cue-induced craving. Correspondingly, we expected that only active stimulation would significantly influence neuronal activity related to the neurocircuitry of craving. Concerning the open-label treatment part, we expected that 15 HF-rTMS sessions would result in significant cue-induced and general craving reductions. We hypothesized that, while performing the cue-exposures inside the fMRI, the expected reductions in cue-induced craving scores would objectively result in a higher impact on the craving neurocircuitry. For both cue-exposure paradigms, we expected similar results except if transient variations in hemodynamic responses are important, then we would expect that only the event paradigm would be sensitive enough to detect them [32].

## Materials and Methods

### Ethics statement

The study was approved by the ethical committee "Commissie Medische Ethiek" of the University Hospital UZBrussel. Written informed consent was obtained from all participants.

### Subjects

The study was carried out over a period of one year (from August 2013 until August 2014 included). All patients (aged between 18 and 65 years) were recruited from our psychiatric ward and all remained hospitalized throughout the entire stimulation protocol. We also evaluated the effect of accelerated HF-rTMS on the relapse neurocircuit. Because these results are beyond the scope of this article, they will be submitted elsewhere.

Alcohol dependence was assessed with the Mini International Neuropsychiatric Interview (Mini) [33], which was administered by the attending physician at the psychiatric ward. Duration of the addiction disorder and number of heavy drinking days (≥5 units/day) the last 30 days before hospitalization were assessed with the Addiction Severity Index (ASI) [34]. The Dutch and French versions of the ASI do not discriminate between gender; therefore ≥5 units/day was used to define heavy drinking for both male and female. Exclusion criteria were left-handedness, current psychotic episode or delirium, any personal history of epilepsy, neurosurgery, the presence of pacemakers or other electronic implants, metal or magnetic objects in the brain, unstable medical condition, pregnancy and cognitive deterioration (Montreal Cognitive Assessment (MoCA) < 26/30 (http://www.mocatest.org/). The co-morbid use of other abusing substances was not an exclusion criterion; however alcohol addiction was the primary diagnosis. If patients were already on anti-craving medication when admitted, a two-week washout period was respected before starting HF-rTMS. The daily routines of the included patients before the start of the HF-rTMS protocol consisted of two-weekly evaluations by their attending psychiatrist. Patients had the freedom of choice to participate in occupational therapy (sports or ergotherapy). Psychotherapeutic interventions were only allowed after the completion of the stimulation protocol.

Thirty-three patients signed written informed consent during the inclusion period. Four patients were considered dropouts before they received HF-rTMS stimulation, because their hospitalization was terminated prematurely. One patient was considered a dropout because she took disulfiram the evening before the start of the protocol. Another patient was a dropout due to movement artifacts. One obese patient was unable to visualize the presented images in the scan because his abdomen blocked the reflection of the pictures, which were presented on a flat screen positioned at the subject’s feet. Two patients dropped out after the completion of the sham-controlled HF-rTMS session: one of them found the active stimulation too painful and the other suffered from too much craving after the completion of the second scan in order to continue. Another patient relapsed immediately after the completion of the treatment protocol but before the final scan could be obtained. Therefore, these three patients were considered dropouts for the treatment part. Of note, two patients were only exposed to the event-related paradigm. Therefore, our final sample for the analysis regarding the event-related paradigm consisted of 26 patients for the experimental part (one placebo-controlled session) and 23 patients for the treatment part (15 active sessions). Concerning the analysis for the block-related paradigm: 24 patients were included for the experimental part and 21 for the treatment part. See Table 1 for the demographic data of the patients.

**Table 1:** Overview of the demographic data.

	All HF-rTMS treated patients (26 patients)	Active HF-rTMS session (13 patients)	Sham HF-rTMS session (13 patients)	*P*-value
Gender (Male/Female)	17/9	9/4	8/5	≥0.99
Age (year)	Total: M = 45.2, SD = 9.3	M = 46.7, SD = 10.4	M = 43.7, SD = 8.1	0.42
	Men: M = 45.1, SD = 10.5			
	Women: M = 45.4,SD = 6.9			
Stimulation depth (mm)	M = 20.5, SD = 2.5	M = 20.3, SD = 2.8	M = 20.6, SD = 2.4	0.77
Benzodiazepine free days before stimulation	M = 14.3, SD = 5.6	M = 14.3, SD = 5.6	M = 10.2, SD = 3.2	0.03
% nicotine	76.9% (20 patients)	11 patients	9 patients	≥0.99
% comorbid drug dependence	15.4% (4 patients)	3 patients (2 patients: cocaine; 1 patient cannabis)	1 patient (cannabis)	0.59
% comorbid benzodiazepine dependence	7.7% (2 patients)	1 patient	1 patient	1.00
Use of anti-craving medication before stimulation (washout of 2 weeks)	2 patients	1 patient	1 patient	1.00
Baseline Alcohol Urge Questionnaire (AUQ)	M = 15.9, SD = 11.0	M = 17.8, SD = 11.4	M = 14.2, SD = 10.8	0.47
Baseline 5-item Obsessive Compulsive Drinking Scale (OCDS)	M = 3.8, SD = 3.8	M = 4.5, SD = 3.7	M = 3.2, SD = 4.0	0.27
Number of days patients drank more than 5 units/day (during the last month before hospitalization) (Alcohol Severity Index (ASI))	M = 19.6, SD = 9.6	M = 16.9, SD = 11.2	M = 22.5, SD = 6.8	0.17
Duration of alcohol addiction (years) (ASI)	M = 12.1, SD = 9.9	M = 13.7, SD = 11.4	M = 10.3, SD = 8.1	0.41
Days between last alcohol intake and the start of the stimulation	M = 17.8, SD = 6.0	M = 19.0, SD = 4.5	M = 16.5, SD = 7.2	0.31

Demographic data concerns the entire patient group. Therefore, the two patients who were not presented with the block-related cue-exposure were also considered. MT: motor threshold; M: mean; SD: standard deviation.

### Detoxification

All patients were detoxified with diazepam according to the Clinical Institute Withdrawal Assessment for Alcohol, Revised (CIWA-AR) [35]. Those patients suffering from a comorbid benzodiazepine addiction (2 patients) received a diazepam substitution scheme that was decreased progressively. All patients were at least one week diazepam-free before the start of the experimental procedure. See Table 1.

### Craving questionnaires

The main outcome measure regarding cue-induced craving were ten-point Likert scales (TLS), administered before and after the block and event-related cue-exposure inside the scanner. TLS was verbally assessed: “On a scale from 0 to 10, how much do you crave for an alcoholic beverage right now?”

To evaluate general craving (craving not related to the cue-exposure paradigm) we used the Alcohol Urge Questionnaire (AUQ) [36] and the Obsessive Compulsive Drinking Scale (OCDS) [37]. The AUQ is an eight-item questionnaire and evaluates three aspects of craving: desire for a drink, expectation of positive effect from drinking and inability to avoid drinking if alcohol was available [38]. The minimum obtainable score is 8, while the maximum obtainable score is 56. Because the OCDS contains items that do not represent the core concept of craving but instead are indicators for the consequences of craving, we used only those items related to subjective craving (items 1, 2, 4, 5 and 13) [39]. The minimum score is 0; the maximum score is 20. Both AUQ and OCDS were administered before the start of the protocol and one week later, before the last scan.

### Study design

**Fig 1:**
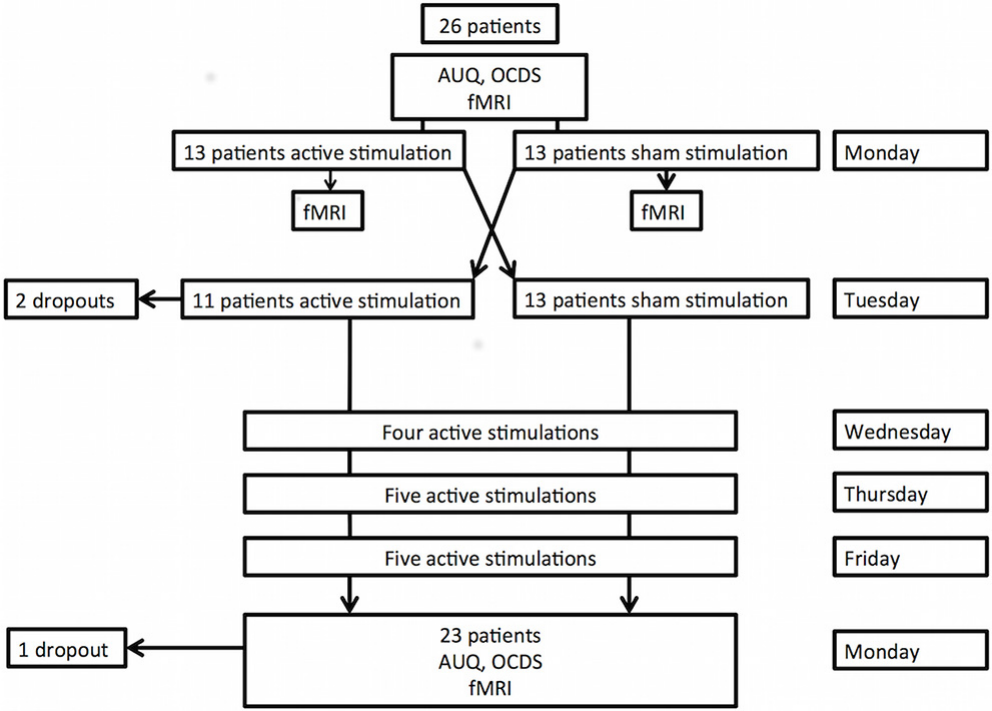
Flowchart of the HF-rTMS treated patients through the study.

The study comprises an experimental part, during which the effect of one single blind right DLPFC sham-controlled HF-rTMS session (between-subjects) is evaluated on alcohol craving and brain activity. The second part consists of an open label treatment study, during which fifteen HF-rTMS sessions are evaluated. For the experimental part patients were randomized into two groups by flipping a coin: one group received active stimulation (13 patients), whereas the other received sham stimulation (13 patients) (Fig 1). All patients and all staff attending to the patients at the unit were blinded for the sham-controlled session. After the completion of the sham-controlled sessions, patients were asked whether they could guess correctly on which day they received the active stimulation. Only 52,2% of the patients guessed correctly the type of stimulation, which is consistent with the study performed by Berlim and colleagues (2013) [40], who state that the existing sham rTMS interventions appear to result in acceptable levels of blinding regarding treatment allocation. The principal investigators were not blinded for the sham-controlled session. Regarding the treatment part, all patients and staff were well aware that only active stimulation was administered.

#### One sham-controlled HF-rTMS session

The stimulation protocol always started on Monday. AUQ and OCDS were administered. Next, during fMRI patients always underwent first a block and second the event-related cue-exposure (see below), during which TLS was verbally assessed before and immediately after both cue-exposures (TLS = > block = > TLS = > event = > TLS, without leaving the scanner). Immediately after the scan, they received the first right DLPFC HF-rTMS session. About twenty minutes (duration of entering the scan and performing the 3D scan) after the stimulation, they were again confronted with a block and event-related cue-exposure in the same order during fMRI. In line with previous neurophysiological and cognitive HF-rTMS research, we expect this time-window as still sufficient to be able to evaluate the effect of one right DLPFC HF-rTMS session [41]. However, at this moment there is no data available regarding the duration of the effect of one HF-rTMS session in alcohol-dependent patients. TLS were assessed the same way.

As mentioned before, for the single sessions patients were randomized to receive one active stimulation session or sham stimulation. The following day they received the other condition: those patients who received active stimulation on Monday were given placebo stimulation on Tuesday and vice versa. Importantly, patients did not perform the fMRI cue-exposures on Tuesday and TLS craving scores were therefore not obtained.

Twenty-six patients completed the experimental part.

#### Accelerated HF-rTMS treatment part

From Wednesday until Friday, all patients received 14 active stimulations: with a between session delay of 15 minutes: 4 on Wednesday, 5 on Thursday and 5 on Friday, making it 15 HF-rTMS sessions in total (23,400 pulses). The next Monday before the start of the last scan, during which patients again were exposed to a block and event-related cue-exposure paradigm, general craving was assessed with the 5-items OCDS and AUQ. Cue-induced craving was also verbally assessed in the scan with TLS (as described above, before and after the presentation of the cue-exposures). Twenty-three patients completed the treatment part.

### HF-rTMS procedure

We used a Magstim high-speed magnetic stimulator (Magstim Company Limited, Wales, UK), connected to a figure-of-eight-formed double 70 mm coil held tangentially to the skull. In order to accurately target the middle of the right DLPFC individually (Brodmann area 9/46), the precise stimulation site and position of the coil was determined using MRI non-stereotactic guidance [42]. In short, we located the right DLPFC visually on the 3D surface rendering of the brain based on the known gyral morphology and marked the middle part of the median prefrontal gyrus as the centre of the right DLPFC (Brodmann 9/46). The corresponding coil position was found by determining the perpendicular projection of this point on the scalp. On the 3D reconstruction of the head we marked four reference points: right ear, left ear, vertex and nose which were connected by two reference axes: one from nose to atlas and one between the two ears. A fifth reference point, ‘‘top” (the projection of the DLPFC on the scalp), was defined by the crossing of the two axes. Perpendicular to this point, the precise stimulation site on the skull was marked and stimulated. The individual resting motor threshold (MT) for the right abductor pollicis brevis muscle was determined using single pulse TMS in combination with motor evoked potentials (MEP). The MT was considered as the lowest intensity to induce a visual MEP on electromyography (EMG) of at least 50 μv in at least 5/10 stimuli. A stimulation intensity of 110% of the subject’s resting MT was used for the study. In each high-frequency (20 Hz) rTMS session, subjects received forty trains of 1.9 s duration, separated by an intertrain interval of 12 s (1560 pulses per session) [22]. For the sham stimulation, the coil was held at an angle of 90°, only resting on the scalp with one edge. Subjects were only kept unaware of the type of stimulation they received during the first two stimulation days; they wore earplugs and were blindfolded.

The study was conducted in conformity with the current safety guidelines [30,43].

### fMRI

The study was carried out on a 3T MRI scanner (Philips Intera, Best, The Netherlands) equipped with an eight-channel sense head coil. We measured 127 consecutive FFE-EPI volumes (TR/TE = 3000/35 ms, flip angle = 90°, 18 slices, slice thickness/gap = 5.0/ 1.0 mm, size = 64 x 64, in plane resolution = 3.75 x 3.75 mm, duration 6 min 21 s) covering the whole brain. During functional magnetic resonance imaging, pictures were back-projected onto a flat screen positioned at the subject’s feet and viewed via a mirror mounted on the head coil. Before the fMRI experiment, a T1- weighted structural scan (3D IR-TFE, TI/TR/TE = 1501/16/4.6 ms, flip angle = 30°, matrix = 256 x 256, in plane resolution = 1.0 x 1.0 mm, 100 slices, slice thickness 2.0 mm, duration 6 min 24 s) of the whole head was performed.

### fMRI designs

As mentioned before, we used both a block and event-related paradigm, administered in this consecutive order. This way, the order of appearance of both paradigms was equal for all patients.

For both paradigms two sets of stimuli were used (alcohol and neutral). The alcoholic stimuli consisted of pictures that differed in alcoholic context (home environment, bar, parties…) and in type of alcoholic beverage (beer, wine, spirits…). The neutral stimuli consisted of pictures of houses. We chose pictures of houses because these pictures differ strongly from the alcoholic pictures and should therefore be assessed as neutral by the alcohol-dependent patients. All pictures were matched for luminescence.

In order to avoid habituation effects, different stimuli were used for both the block and the event cue-exposure paradigms. Therefore, because patients were scanned three times, three different blocks and three different events were created and presented. We randomized the sequence of the paradigms over the three scan moments.

#### Block-related cue-exposure (Fig 2A)

Blocks were randomly presented. For each condition (alcohol–neutral) they were 7 blocks, each consisting of 5 stimuli. The stimuli were presented for 3 seconds, with an inter stimulus interval of 1s. For attentional purposes, eight of these fourteen blocks (4 alcohol and 4 neutral blocks) were followed with a central yellow circle, depicted for 1s; the moment this yellow circle was presented patients had to press a response button as quickly as possible. Each block was followed by a 16s rest period (white fixation cross on a black screen), or 15 s when the block was followed by a yellow circle. The total duration of the block paradigm was 525 s.

**Fig 2:**
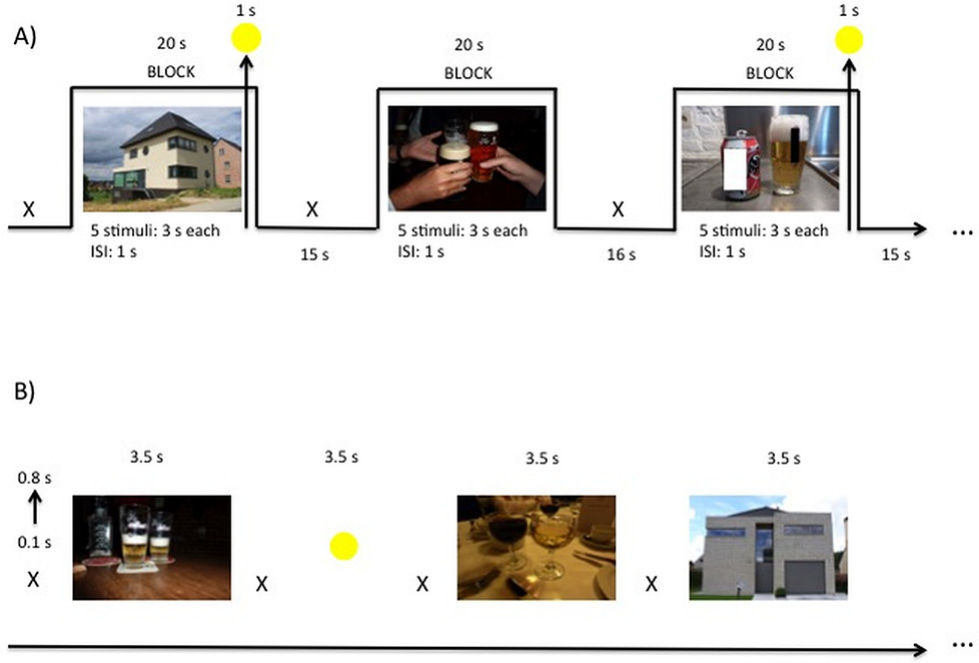
(A) Block-related and (B) event-related cue-exposure paradigm during fMRI. The white fixation cross was jittered with a duration between 0.1 and 0.8 seconds. TLS were verbally assessed before and after the block-related cue-exposure and after the event-related paradigm. s = seconds.

#### Event-related cue-exposure (Fig 2B)

The 48 alcohol-related and 48 neutral stimuli were presented randomly. They were displayed for 3.5 seconds and preceded by a jittered white fixation cross, with duration of minimum 0.1 seconds and maximum 0.8 seconds, centered on a black background. Comparable with the block paradigm, yellow circles (15 in total), were projected on a black background and mingled between the alcoholic and neutral pictures for attentional purposes. The total duration of the event paradigm was 465 s.

### Statistical analysis—Craving data

All collected craving data were analyzed with SPSS 22 (Statistical Package for the Social Sciences). The significance level was set at *P*<0.05, two-tailed for all analyses. Normality was assessed with the Kolmogorov Smirnov test.

#### One sham-controlled HF-rTMS session

To evaluate the experimental part, we took 6 TLS scores into account: TLS 1, TLS 2 and TLS 3 before the stimulation session (TLS 1 = before the cue-exposures; TLS 2 = after the block-related cue-exposure and TLS 3 = after the event-related cue-exposure); and TLS 4, TLS 5 and TLS 6 after the stimulation session (TLS 4 = before the cue-exposures; TLS 5 = after the second block-related cue-exposure and TLS 6 = after the second event-related cue-exposure).

TLS scores were not normally distributed (all *P*’s<0.05). Transforming in order to normalize the data was unsuccessful. Therefore, to evaluate the single session non-parametric tests (Friedman Test, Wilcoxon Signed Rank Test, and Mann-Whitney U Test) were used.

#### Accelerated HF-rTMS treatment part

In order to evaluate accelerated HF-rTMS treatment outcome, we considered the AUQ, OCDS and all TLS of the first and last (TLS 7 = before the cue-exposures, TLS 8 = after the third block-related cue-exposure and TLS 9 = after the third event-related cue-exposure) scan. Due to non-normality (all *P*’s<0.05), we again used non-parametric tests (Friedman, Wilcoxon Signed Rank Test and Mann-Whitney U).

### Statistical analysis—Imaging data

The fMRI data were analyzed with SPM8 software (Wellcome Department of Cognitive Neurology, London, UK). The fMRI time series were realigned to their first volume to correct for head movements. For the block-related design, a realignment step, normalization into the standard anatomical space (EPI MNI template) and smoothing with an 8 mm Gaussian kernel were performed. For the event-related design, also a slice timing correction was performed between the realignment and the normalization step in order to correct for the difference in time points between consecutive slices for each scanned brain volume. The anatomical scan was normalized to the standard anatomical space (T1 MNI template) to be used as anatomical underlay for the results. For the block-related design, we modeled our three regressors of interest, alcohol, neutral and circle, as separate boxcar functions convolved with the canonical HRF. For the analysis of the event-related design, the three regressors were also convolved with the time-derivative of the canonical HRF to correct for deviations between the real HRF and the theoretical HRF. For both designs we added to the regression model six motion regressors (3 translation, 3 rotation) to take residual motion into account and a constant to model the activation onset. This model was fitted to the measured data using the generalized linear model (GLM) approach. For each subject, we generated contrast (percentage signal change) maps and t-statistic maps corresponding to the contrast alcohol versus neutral.

Corresponding brain regions were identified with the Talairach Daemon (search range: 5 mm) of the SPM implemented WFU PickAtlas [44,45,46,47].

#### Baseline scan–Block-related cue-exposure paradigm

Starting from the individual contrast maps, we performed a one-sample *t*-test for the contrast *alcohol* versus *neutral*, using the first (baseline) scan from each individual before entering the HF-rTMS protocol. Age and gender were covariates. Results are reported at a voxel height threshold at *P*<0.001 significance, conducted with an AlphaSim correction [48], as implemented in the REST toolbox (restfmri.net/forum/), bringing forth a minimum cluster extent threshold (k) of 260 at *P*<0.05. To evaluate a possible correlation between TLS and the neural responses to the alcohol-related stimuli, we performed a regression analysis at a voxel height threshold at *P*<0.001 significance based on the individual response maps for the block-related cue-exposure with TLS scores obtained immediately after the block paradigm as a regressor (minimum k of 164 after AlphaSim correction at *P*<0.05). A constant was included in the regression to model the mean neural response.

#### Baseline scan–Event-related cue-exposure paradigm

As for the block-related paradigm, we performed a one-sample *t*-test for the contrast *alcohol* versus *neutral* using the baseline scan from each individual. Results are reported at a voxel height threshold at *P*<0.001 significance, with a minimum k of 296 after AlphaSim correction at *P*<0.05. Comparable with the block-related cue-exposure, a regression analysis was performed with TLS scores obtained after the event-related cue-exposure as a regressor (minimum k of 211 after AlphaSim correction at *P*<0.05).

#### One sham-controlled session–Block-related cue-exposure paradigm

To investigate the effect of one HF-rTMS session on brain activity, we performed a full factorial group analysis. Within-subject variable was TIME (before versus after HF-rTMS). Between-subject variable was STIMULATION (active versus sham), we selected only those TIME x STIMULATION interaction clusters with a voxel height threshold at *P*<0.005 significance and minimum k of 55 after AlphaSim correction at *P*<0.05,

To investigate whether the significant clusters corresponded to increased or decreased neuronal activity, in a following step, post hoc paired *t*-tests were performed with the interaction mask of the full factorial group analysis at *P*<0.005.

#### One sham-controlled session–Event-related cue-exposure paradigm

Similar to the analysis of the block-related paradigm, results are reported at a voxel height threshold at *P*<0.005 significance, with a minimum k of 59 after AlphaSim correction at *P*<0.05.

#### Accelerated HF-rTMS treatment part–Block-related cue-exposure paradigm

To investigate the effect of 15 accelerated HF-rTMS sessions on brain activity, a paired *t*-test was performed with a voxel height threshold at *P*<0.005 significance, with a minimum k of 356, after AlphaSim correction at *P*<0.05

#### Accelerated HF-rTMS treatment part–Event-related cue-exposure paradigm

Comparable with the analysis of the block-related cue-exposure, a paired *t*-test was performed with a voxel height threshold at *P*<0.005 significance, with a minimum k of 425, after AlphaSim correction at *P*<0.05.

## Results

### Craving assessment

#### One sham-controlled HF-rTMS session

First of all, we observed no group differences (between the group receiving first active versus first sham HF-rTMS) in demographic data, such as age (t(24) = 0.82, *P* = 0.42), gender distribution (Fisher’s exact test: *P*>0.99), stimulation depth (t(24) = -0.30, *P* = 0.77), duration of the addiction disorder (t(21) = 0.84, *P* = 0.41, number of heavy drinking days (≥5units/day) the last 30 days before hospitalization (t(21) = 1.42, *P* = 0.17) and alcohol free days before stimulation (t(24) = 1.05, *P* = 0.31). Because of amnestic difficulties, there were two missing values for the severity of the alcohol dependence and for the duration of the alcohol problem. There was a significant difference in benzodiazepine free days before the start of the stimulation (t(24) = 0.19, *P* = 0.03). However, all patients were at least 7 days benzodiazepine free at the start of the protocol (active stimulation: M = 14.3 days, SD = 5.6; sham stimulation: M = 10.2, SD = 3.2).

For an overview of TLS craving scores see Table 2. All TLS were assessed on a range from 0 to 10. There were two missing TLS craving scores after the block-related cue-exposure, because two patients were only exposed to the event-related paradigm. The Friedman Test did not show a significant effect on TLS-scores for the active stimulation (*X* ^*2*^(5) = 3.56, *P* = 0.61) and the sham stimulation (*X* ^*2*^(5) = 4.51, *P* = 0.48). Further, the Mann-Whitney U test did not show a significant difference in TLS 1, 2, 3, 4, 5 and 6 (all *P*'s > 0.05) between both stimulation groups. Because previous treatment with anti-craving medication (2 patients in total, 1 in each group) could have influenced craving measures at baseline, we also performed a Mann-Whitney U test for TLS 1, 2 and 3 between both groups omitting the two patients where an anti-craving medication washout period of 2 weeks had been respected. This demonstrated no significant differences (all *P*'s >0.05).

**Table 2:** Overview of the TLS craving scores.

	Before HF-rTMS			After one HF-rTMS session			All patients		
							After accelerated HF-rTMS treatment		
Patients receiving:	TLS 1	TLS 2	TLS 3	TLS 4	TLS 5	TLS 6	TLS 7	TLS 8	TLS 9
Active stimula-tion	M = 2.3	M = 2.1	M = 2.6	M = 1.8	M = 1.9	M = 2.2	M = 1.0	M = 1.0	M = 1.1
	SD = 2.6	SD = 2.8	SD = 2.8	SD = 2.1	SD = 2.1	SD = 2.1	SD = 1.8	SD = 2.1	SD = 2.0
	Range = 8.0	Range = 9	Range = 9	Range = 7	Range = 7	Range = 7	Range = 7	Range = 8	Range = 8
	Median = 2.0	Median = 1.0	Median = 3.0	Median = 1.0	Median = 2.0	Median = 2.0	Median = 0.0	Median = 0.0	Median = 0.0
	25-perc = 0.0	25-perc = 0.0	25-perc = 0.0	25-perc = 0.0	25-perc = 0.0	25-perc = 0.0	25-perc = 0.0	25-perc = 0.0	25-perc = 0.0
	75-perc = 4.5	75-perc = 3.0	75-perc = 3.5	75-perc = 3.0	75-perc = 3.0	75-perc = 3.5	75-perc = 2.0	75-perc = 1.0	75-perc = 1.0
Sham stimula-tion	M = 2.0	M = 2.2	M = 2.5	M = 1.8	M = 2.0	M = 2.2			
	SD = 2.8	SD = 2.9	SD = 3.1	SD = 2.4	SD = 3.1	SD = 3			
	Range = 8.5	Range = 9	Range = 9	Range = 8	Range = 9	Range8 =			
	Median = 1.0	Median = 0.0	Median = 1.0	Median = 1.0	Median = 0.0	Median0.0 =			
	25-perc = 0.0	25-perc = 0.0	25-perc = 0.0	25-perc = 0.0	25-perc = 0.0	25-perc = 0.0			
	75-perc = 3.0	75-perc = 5.0	75-perc = 5.0	75-perc = 3.0	75-perc = 5.0	75-perc = 5.0			
Total	M = 2.1	M = 2.2	M = 2.5						
	SD = 2.6	SD = 2.8	SD = 2.9						
	Range = 8.5	Range = 9	Range = 9						
	Median = 1.0	Median = 0.5	Median = 2.0						
	25-perc = 0.0	25-perc = 0.0	25-perc = 0.0						
	75-perc = 3.3	75-perc = 3.8	75-perc = 4.3						

TLS was assessed on a score from 0 to 10. TLS 1 -> TLS 6: craving scores before and after one sham-controlled HF-rTMS session. TLS 1: before the start of the protocol; TLS 2: after the first **block** cue-exposure; TLS 3: after the first **event** cue-exposure; TLS 4: before the second cue-exposures. TLS 5: after the second **block** cue-exposure; TLS 6: after the second **event** cue-exposure

TLS 7 -> TLS 9: craving scores after 15 accelerated HF-rTMS sessions (all patients). TLS 7: before the cue-exposures; TLS 8: after the third **block** cue-exposure; TLS 9: after the third **event** cue-exposure. M = Mean; SD = Standard deviation; Perc = percentile

To evaluate whether our sample was not underpowered, we performed a power analysis with G*power [49]. Because it is not possible to perform a power analysis for non-parametrical tests in a 6x2 design, power was assessed for an ANOVA with 6 measurements and 2 groups, assuming medium effect size *f* of 0.25, which revealed a total sample size of 20. The actual effect size cannot be calculated because previous research concerning the effect of one HF-rTMS session on TLS during a cue-exposure is non-existent.

#### Accelerated HF-rTMS treatment part

There were missing values for the OCDS before the first scan of two patients. Three patients found the AUQ too difficult to complete. Therefore, the OCDS was considered for twenty-one patients, while the AUQ was considered for twenty patients. All twenty-three patients were considered for the analysis of TLS. See also Table 2 for the demographic data of the TLS craving scores and Table 3 for the demographic data of the OCDS and AUQ. The Wilcoxon Signed Rank Test showed significance for both the OCDS (Z = -2.41; *P* = 0.02) and the AUQ (Z = -2.36; *P* = 0.02) after HF-rTMS treatment, meaning that OCDS and AUQ scores, which were obtained outside the scanner, decreased after accelerated HF-rTMS treatment. For two patients there were missing values for the TLS immediately after the block-related cue-exposure paradigm, because those patients were only exposed to the event-related paradigm. For an overview of TLS craving scores see Table 2. The Friedman Test showed a significant effect on TLS-scores (X^2^(5) = 24.32, *P* = 0.001). Post-hoc Wilcoxon Signed Rank tests showed a significant effect between all TLS of the first scan compared with all TLS of the last scan (all *P*'s < 0.05). However, all other TLS comparisons were not significant (all *P*'s > 0.05). Therefore, craving changes only appeared in the time-period between the first and final scan-moments and not during the presentation of the cue-exposures.

**Table 3:** Craving data confined to the accelerated HF-rTMS treated patients for the Obsessive Compulsive Drinking Scale (OCDS) and the Alcohol Urge Questionnaire (AUQ).

HF-rTMS treated patients	OCDS	AUQ
	M: 3.95	M: 16.85
Before treatment	SD: 9.90	SD: 11.53
	Median: 3.00	Median: 12.50
	Range: 12.00	Range: 38.00
	25-percentile: 0.00	25-percentile: 8.00
	75-percentile: 8.00	75-percentile: 23.00
	M: 2.43	M: 13.20
After treatment	SD: 2.56	SD: 7.98
	Median: 2.00	Median: 8.50
	Range: 9.00	Range: 28.00
	25-percentile: 0.00	25-percentile: 8.00
	75-percentile: 3.5	75-percentile: 19.00

M = mean; SD = standard deviation

### Imaging Data

#### Baseline scan–Block-related cue-exposure paradigm

The scans of twenty-four patients were considered.

The alcohol > neutral contrast yielded one activated cluster, the left inferior frontal gyrus (MNI coordinates: x = -42, y = 22, z = 0). See also Table 4. The regression analysis showed no meaningful correlations.

**Table 4:** Results of the random effect analysis for the contrast *alcohol* versus *neutral* at baseline.

fMRI design	Cluster size	Anatomical region	hemisphere	BA	Peak	Peak	Peak
					T-value	Z-value	Coordinates
T-contrast *alcohol* versus *neutral*							
BLOCK	841	inferior frontal gyrus	left	47	6.26	4.65	-42 22 0
EVENT	804	Middle Occipital Gyrus	Right		10.8	6.38	46–68–6
	2551	Inferior Parietal Lobule	Left	-	9.29	5.93	-32–54 48
	675	Parietal Lobe, sub-gyral	Right	7	8.07	5.50	28–56 38
	814	Inferior Frontal Gyrus	Left	-	8.00	5.48	-32 32–12
	1577	Inferior Frontal Gyrus	Left	47	7.39	5.24	-46 6 24
	433	Cuneus	Right	18	7.18	5.15	10–96 8
	1520	Medial Frontal Gyrus	Right	8	6.96	5.06	4 22 52
	641	Precentral Gyrus	Right	6	6.00	4.61	38–4 44

We listed only those clusters with a significance of *P*<0.001. For each cluster, we reported the T-value, Z-value and MNI coordinates at the position of the maximum, the cluster size and the appropriate corresponding Brodmann Area (BA).

#### Baseline scan–Event-related cue-exposure paradigm

The scans of twenty-six patients were considered.

**Fig 3:**
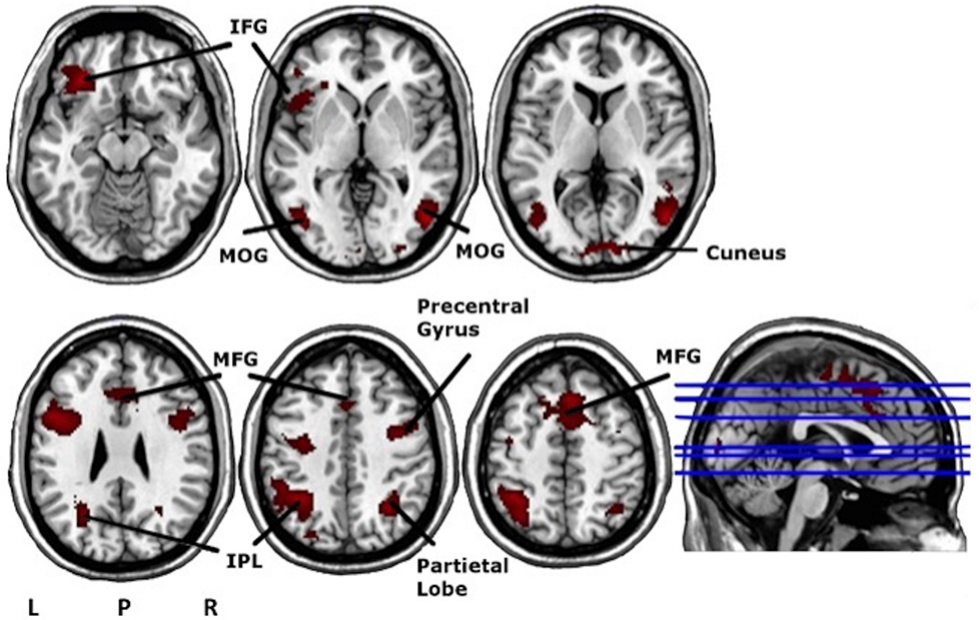
Axial and sagittal views of the significant clusters of the random effects analyses during the event-related cue-exposure (contrast *alcohol* versus *neutral*), overlaid on an anatomical T1-image. These analyses were performed at a voxel height threshold at *P*<0.001 significance (minimum K = 296). IFG = Inferior Frontal Gyrus; MOG = Middle Occipital Gyrus; IPL = Inferior Parietal Lobule; MFG = Medial Frontal Gyrus; L = left; R = right; P = posterior.

The *alcohol > neutral* contrast yielded eight major clusters, among the left inferior frontal gyrus (BA 47: MNI coordinates: x = -32, y = 32, z = -12) (See also Table 4 and Fig 3). Also here, the regression analysis showed no meaningful correlations.

#### One sham-controlled session–Block-related cue-exposure paradigm

The scans of twenty-four patients were considered.

The interaction effect TIME *X* STIMULATION did not yield any activated clusters. Therefore, post hoc tests were not performed.

#### One sham-controlled session–Event-related cue-exposure paradigm

The scans of twenty-six patients were considered.

**Fig 4:**
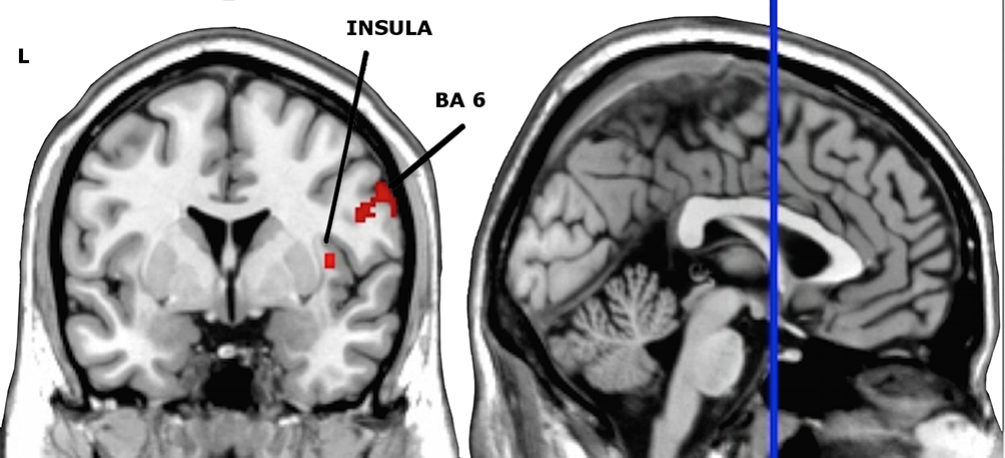
Coronal and sagittal views of the significant clusters of the post hoc paired *t*-tests for the active stimulation with the interaction mask of the full factorial at *P*<0.005, overlaid on an anatomical T1 image. The red color represents an increase in neural activation after active stimulation. Cluster sizes (AlphaSim corrected): Insula = 49, BA6 = 68. L = left; BA = Brodmann Area.

This interaction effect *TIME X STIMULATION* produced 2 clusters reaching significance in the right hemisphere, among the right inferior frontal gyrus (BA 9: MNI coordinates: x = 48, y = -2, z = 24). Post hoc paired *t*-tests showed that one right-sided active HF-rTMS session enhanced activity in the right insula (MNI coordinates: x = 38, y = -2, z = 8) and in the right precentral gyrus (BA6: MNI coordinates: x = 50, y = -2, z = 26). See Table 5 and Fig 4. Post hoc paired *t*-tests showed that one right-sided sham HF-rTMS session decreased activity in the insula (MNI coordinates: x = 38, y = 14, z = -2). See also Table 5.

**Table 5:** Results of the full factorial analysis for the contrast *alcohol* versus *neutral* and post hoc brain imaging results of the single placebo-controlled HF-rTMS session.

Cluster size		Anatomical region	hemisphere	BA	Peak	Peak	Peak
					F-value	Z-value	coordinates
Main effect of TIME (before and after HF-rTMS): F-contrast *alcohol versus neutral*							
**Block:**							
No Significant clusters emerged							
**Event:**							
110		Lateral ventricle	Right	-	16.72	3.58	4–16 20
160		Putamen	Right	-	13.94	3.28	24 16–8
86		Inferior Frontal Gyrus	Left	47	11.60	2.99	-28 28–4
Main effect of STIMULATION (active versus sham HF-rTMS): F-contrast alcohol versus neutral							
**Block:**							
No Significant clusters emerged							
**Event:**							
No significant clusters emerged							
Interaction between TIME (before and after HF-rTMS) and STIMULATION (active versus sham):							
F-contrast alcohol versus neutral							
**Block:**							
No Significant clusters emerged							
**Event:**							
131		Insula	Right	-	14.48	3.34	40 4 4
100		Inferior Frontal Gyrus	Right	9	13.46	3.22	48–2 24
Post hoc paired t-tests	Cluster size	Anatomical region	hemisphere	BA	Peak	Peak	Peak
**only performed for the EVENT paradigm**					F-value	Z-value	coordinates
**Active HF-rTMS**		No significant clusters					
Pre>Post		emerged					
Post>Pre	49	Insula	Right	-	3.83	3.55	38–2 8
	68	Precentral Gyrus	Right	6	3.31	3.12	50–2 26
**Sham HF-rTMS**							
Pre>Post	18	Insula	Right	13	3.31	3.12	38 14–2
Post>Pre		No significant clusters					
		emerged					

For the interaction effects, we listed those clusters that reached significance with a *P*<0.005 AlphaSim corrected voxel threshold. For each cluster we reported the F-value, Z-value and MNI coordinates at the position of the maximum, the cluster size and the appropriate Brodmann Area (BA). For each significant cluster of the post hoc results, we reported the T-value, Z-value and MNI coordinates at the position of the maximum, the cluster size and the appropriate BA.

#### Accelerated HF-rTMS treatment part–Block-related cue-exposure paradigm

The scans of twenty-one patients were considered.

No significant change was brought about by 15 accelerated HF-rTMS sessions, while patients were being exposed to the block-related cue-exposure paradigm.

#### Accelerated HF-rTMS treatment part–Event-related cue-exposure paradigm

The scans of twenty-three patients were considered.

**Fig 5:**
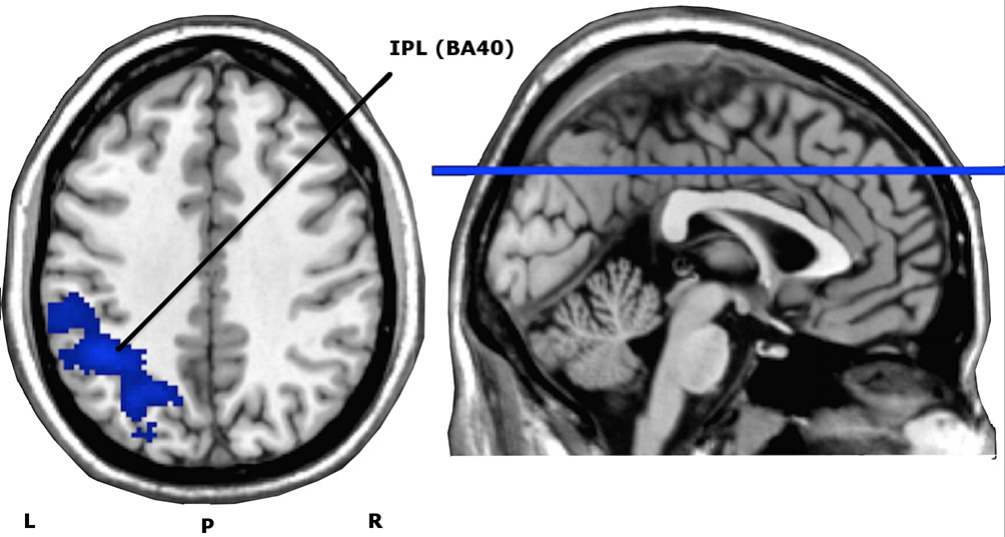
Axial view of whole brain activity found with the paired *t*-test (contrast Before > After HF-rTMS treatment), overlaid on an anatomical T1-image. The depicted blue area represents the significantly deactivated cluster for a voxel significance threshold of *P*<0.005. IPL = Inferior Parietal Lobule (cluster size (AlphaSim corrected): 1141). L = left; R = right; P = posterior; BA = Brodmann Area.

Paired *t*-test demonstrated a significant decrease in neuronal activity after fifteen active right DLPFC HF-rTMS sessions in the left inferior parietal lobule (IPL) (BA 40: MNI coordinates: x = -44, y = -50, z = 40), with a cluster size of 1141, peak T-value of 5.09 and peak Z-value of 4.09. There were no brain areas that increased in neuronal activation after fifteen active treatment sessions. See Fig 5.

## Discussion

To our knowledge, this is the first study that evaluates the effect of a single (sham-controlled) and multiple (open) accelerated HF-rTMS sessions on brain activity in detoxified alcohol-dependent patients during a block and event-related alcoholic cue-exposure paradigm. Overall, stimulation was well tolerated. Only two patients complained of a post-stimulation headache that subsided with the administration of an oral analgesic.

### Discussion confined to baseline cue-exposure

When comparing the contrast alcohol versus neutral, both block and event-related cue-exposures elicited neuronal activity changes in the inferior frontal gyrus (BA 47), part of the lateral OFC. The involvement of the OFC in craving, motivation and reward assessment is not unexpected [7,50]. Furthermore, for the event-related paradigm, in line with other cue-induced alcohol-related paradigms, we observed neuronal activity in the middle occipital gyrus, the inferior parietal lobule, cuneus and medial frontal gyrus [23].

However, not in line with our expectations, cue-induced alcohol craving was not altered and remained low. Also, our regression analysis showed no correlation with the craving neurocircuit. Here, we have to note that for our study our recently detoxified alcohol-dependent patients were still hospitalized and were not allowed to consume alcohol afterwards; which to some extent may explain the lack of induced alcohol craving [25]. It has been documented that alcohol craving is often denied by detoxified alcohol-dependent patients [5]. Alternatively, when exposed to the alcohol-related stimuli non-conscious appetitive reactions may have occurred, which is partly supported by our fMRI findings on increased salience attribution [4,5,8].

### Discussion confined to the single session

One active HF-rTMS session did neither significantly change cue-induced craving nor did it alter craving related neurocircuit neuronal activity. However, for the event-related design, one active HF-rTMS session increased activity in the right insula and the right BA 6. The insula is engaged in the processing of external salient stimuli [51,52,53] and often correlates with enhanced cue-induced conscious craving [54]. Because its importance in physiological arousal and the awareness of different kinds of emotions, we cannot rule out that performing the cue-exposure paradigm resulted in other emotional changes rather than craving [55]. The insula has interoceptive functions and because of its reciprocal connections with ACC, ventromedial PFC, amygdala and ventral striatum, it integrates autonomic and visceral information with emotion and motivation [54]. On the other hand, the involvement of the insula has not been consistently observed in alcohol-craving patients [56]. Interestingly, the insula—in line with its role in salience detection—shows transient hemodynamic responses when patients are confronted with emotional stimuli, which are especially detected by using an event-related design [32].

We also observed increased neuronal activity in the right BA 6 after viewing alcohol-related cues as indeed drug cues often result in increased activity in these regions [4,23]. This region consists of the premotor cortex and supplementary motor area (SMA) and is part of an extensive neurocircuit, that stores and processes action knowledge and tool use skills, drug-taking skills included [57].

### Discussion confined to the treatment part

As expected, general craving (assessed with the AUQ and OCDS) decreased after 15 sessions spread over a 4-day period. Although these stimulation results are in line with the work of Mishra and colleagues (2010) [17] who also observed a decrease of general alcohol craving after 10 daily active right DLPFC HF-rTMS sessions, these results are indicative because time effects cannot be excluded due to the absence of a sham-group. However, performed three days after the last stimulation, cue-induced alcohol craving measurements were not influenced (TLS scales). Furthermore, 15 active HF-rTMS sessions did not significantly affect the craving neurocircuit, for neither the block nor the event-related paradigm.

We found only during the presentation of the event-related cue-exposure paradigm a decreased activity in the left inferior parietal lobule (IPL) after HF-rTMS treatment. This parietal region is part of the neurocircuit involved in approach-related actions toward drug stimuli (drug-taking skills) and in the sensory representation of drug cues [58]. Also, the IPL is part of the default mode network (DMN) [59], which is activated in internally directed cognitive operations. It has been demonstrated that increased DMN activity disrupts attention and may be related to increased craving experience and ruminative thoughts about using drugs [60]. In addition, hyperactivation of parietal regions is often observed during exposure to drug-related cue-reactivity paradigms, indicating increased salience attribution [57]. Therefore, in spite that cue-induced craving and the related craving neurocircuit—as proposed by Koob and Volkow (2010) [7]—was not affected, the observed attenuation of IPL activation by right DLPFC HF-rTMS treatment may suggest a decrease of sensory and action-related processing of alcoholic stimuli.

### Limitations

Besides the relatively small sample size, some study limitations have to be discussed. Patients were hospitalized and were well aware of the fact that they could not obtain alcohol during the entire stimulation protocol, which could have influenced our craving induction significantly. It should be noted that before the start of the accelerated treatment protocol patients already scored relatively low on craving (AUQ, OCDS, and TLS), despite their baseline scan indicated increased salience attribution to the alcohol-related stimuli. Wilson et al (2004) [25] point out the fact that treatment-seeking patients not often experience or acknowledge craving when confronted with an alcohol-related cue-exposure. Knowing they would not be rewarded with alcohol consumption after the experiments might explain to some extent the lack of increased subjective alcohol craving and why HF-rTMS did not change neuronal activity in areas related to the craving neurocircuit [61]. Further, the lack of a placebo group for the treatment part should be considered as a limitation of this study, because we cannot exclude a possible time-effect on craving and imaging data. Due to the new and exploratory nature of the study protocol, the only part that was feasible to sham-control was the first stimulation session. We assumed that when patients completed their sham-controlled session, therefore having received both a sham and an active stimulation, this would have increased chances that patients were aware to which stimulation they were allocated to for the treatment sessions. Therefore, we chose to administer only active stimulation in all our included patients, in spite that the single session was not found to suffer from blinding issues. Future studies however may do well to include a sham treatment group. Because we assessed our patients three days after stimulation, it is possible that due to the lack of a longer follow-up period, delayed HF-rTMS effects on cue-induced craving were missed. This is especially important because we used an accelerated HF-rTMS protocol, of which the effect latency in alcohol-dependent patients is not fully understood. Also, habituation processes due to multiple exposures to the stimuli cannot be excluded. Lastly, it is unclear why the block-related cue-exposure in contrast to the event-related paradigm did not elicit brain activity changes after HF-rTMS. At baseline, when exposed to the alcohol-related cues the block-related paradigm elicited less neuronal activity in brain areas implicated in salience attribution compared to the event-related paradigm. When patients are frequently exposed with alcohol-related cues in a short time-frame the event-related paradigm could be more sensitive to detect neuronal changes. Also, it is possible that mainly changes in transient hemodynamic responses occurred, rendering the event-related cue-exposure superior compared to the block-related paradigm. These transient hemodynamic responses are especially known for the anterior insula [32]. Because the block-related paradigm was always followed by the event-related paradigm, possible carry-over effects were the same for all patients.

### Conclusions

Although the lack of a placebo-arm may limit firm conclusions, our findings suggest that accelerated HF-rTMS treatment positively affects general craving in recently detoxified patients (AUQ and OCDS). However, when such patients were exposed to an alcohol-related cue-exposure, after one or after fifteen HF-rTMS sessions, this did not significantly affect the underlying craving neurocircuit. On the other hand, both one HF-rTMS session and the accelerated treatment protocol had an impact on the extended reward neurocircuit, in particular on salience and attention networks. However, only when assessed with an event-related cue-exposure. Especially the effect of 15 accelerated HF-rTMS sessions on the neurocircuit may indicate a decrease in attention directed at alcoholic stimuli. In line with this assumption, Vanderhasselt et al (2007) [62] previously showed beneficial effects of right DLPFC HF-rTMS stimulation on attentional processes in healthy subjects. Besides placebo-controlled research, it might be worthwhile to examine the effect of daily stimulation on the craving neurocircuit following a more classic stimulation protocol, addressing lateralization effects.
